# A novel two-dimensional transition metal dichalcogenide as water splitting photocatalyst with excellent performances

**DOI:** 10.3389/fchem.2022.1003027

**Published:** 2022-08-25

**Authors:** Fang Wang, Zishuang Cheng, Xiaoming Zhang, Chunxiao Xie, Fucai Liu, Chuntao Chang, Guodong Liu

**Affiliations:** ^1^ School of Mechanical Engineering, Neutron Scattering Technical Engineering Research Center, Dongguan University of Technology, Dongguan, China; ^2^ School of Optoelectronic Science and Engineering, University of Electronic Science and Technology of China, Chengdu, China; ^3^ School of Materials Science and Engineering, Hebei University of Technology, Tianjin, China; ^4^ Guangdong-Taiwan College of Industrial Science & Technology, Dongguan University of Technology, Dongguan, China

**Keywords:** two-dimensional materials, transition metal dichalcogenides, water splitting photocatalyst, high mobility, density functional theory

## Abstract

With the rising demand for renewable energy, photocatalysts are considered the most promising solution to harness solar energy, and the search for photocatalysts with excellent performances remains an urgent task. Here, based on density functional theory (DFT), the photocatalytic properties of MoWS_4_ are systematically investigated. The MoWS_4_ monolayer and bilayer are demonstrated as semiconductors with indirect band gaps of 2.01 and 1.48 eV. Moreover, they exhibit high and anisotropic light absorption coefficients of up to ∼10^5^ cm^−1^ in the visible-ultraviolet region. The intrinsic band edge positions could fully satisfy the redox potentials of water without any external adjustment. The electron mobility of MoWS_4_ monolayer is 557 cm^2^ V^−1^s^−1^, which is seven times higher than MoS_2_ monolayer. Hence, MoWS_4_ can be regarded as a promising 2D photocatalyst candidate for water splitting.

## Introduction

With the depletion of fossil energy and the increasing pollution of the natural environment, the demands for renewable energy become critical and urgent for sustainable development of global economy. In 1972, Fujishima and Honda discovered that TiO_2_ can split water to produce hydrogen and oxygen in the presence of sunlight, making photocatalysis one of the most noteworthy solutions to harness solar energy (Fujishima and Honda, 1972). Afterwards, great efforts have been made to develop effective photocatalysts, including transition metal oxides, sulfides, nitrides, and so forth (Tsuji et al., 2005; Suntivich et al., 2011; Han et al., 2018). However, the low quantum efficiency derived from charge recombination on the surface and in the bulk of these photocatalysts could not meet the criteria of favorable photocatalyst for sunlight driven water splitting (Fujishima et al., 2000).

Due to the interesting structures and corresponding electronic properties, two-dimensional (2D) materials have been widely used in various fields and also provide new research directions for efficient photocatalysis (Singh et al., 2015). In recent years, 2D photocatalysts showed the greater advantages over their bulk phase counterparts in terms of photocatalytic performance. For example, Both SnS_2_ monolayer and ZnSe nanosheet exhibited higher photocurrent density than their bulk materials (Sun et al., 2012a; Sun et al., 2012b). In addition, various 2D materials have also been theoretically and experimentally demonstrated to be used as photocatalysts for water splitting, such as 2D transition metal dichalcogenides, g-C_3_N_4_, phosphorene, and so on (Wang et al., 2009; Zhuang and Hennig, 2013a; Rahman et al., 2016; Phuc et al., 2018; Zhang et al., 2022). However, few of these photocatalysts can simultaneously satisfy high visible light absorption, high carrier mobility and perfect band edge positions. For instance, MoS_2_ monolayer showed lower carrier mobility, and MoTe_2_ monolayer can’t perfectly meet the redox potential of water (Wang et al., 2009; Zhuang and Hennig, 2013a; Cai et al., 2014; Rahman et al., 2016; Zhang et al., 2022). Both GaS and GaSe monolayers demonstrated low visible light absorption due to the large band gaps (Zhuang and Hennig, 2013b). Therefore, it is still a challenge to develop water splitting photocatalysts with excellent performances.

In this work, based on the first-principles calculations, we propose a novel 2D transition metal dichalcogenide namely MoWS_4_ and systemically investigate its photocatalytic properties. Firstly, the stability of MoWS_4_ monolayer is confirmed by calculating its phonon spectra and ab initio molecular dynamics (AIMD) simulations. Secondly, we calculate the band structures of MoWS_4_ monolayer and bilayer, and show their semiconductive characteristic. Then, relevant photocatalytic properties are systematically investigated. It is found that MoWS_4_ monolayer and bilayer can nicely meet the redox potentials without strain engineering, and their light absorption coefficients reach ∼10^5^ cm^−1^ in the visible-ultraviolet region. Moreover, the electronic mobility of MoWS_4_ monolayer is as high as 557 cm^2^ V^−1^s^−1^.

## Computational details

For geometric and electronic structures, all calculations are performed by using the Vienna ab into simulation package (VASP) based on density functional theory (DFT) (Kresse and Furthmuller, 1996). We choose the generalized gradient approximation (GGA) of the Perdew–Burke–Ernzerhof (PBE) functional as the exchange-correlation functional to perform these calculations (Perdew et al., 1996). For the 2D monolayer structure, the vacuum layer thickness is set as about 20 Å to avoid layer-to-layer effects. The kinetic energy cutoff is set as 500 eV. The Brillouin zone is regulated with 10 × 6 × 1. During the calculations, the DFT-D2 method with Grimme correction is used to describe the long-range van der Waals interactions (Grimme, 2006). Besides, all atoms are fully relaxed, and the energy and force convergence criteria are set as 10^–6^ eV and 0.01 eV Å^−1^, respectively. Except for PBE functional, to obtain the more accurate results, the HSE06 functional is also adopted to calculate the band structures and the band edge positions (Deák et al., 2010). To identify structural stability of MoWS_4_ monolayer, its phonon spectra are calculated by using the PHONOPY code (Gonze and Lee, 1997). A 6 × 3 supercell structure of MoWS_4_ monolayer is used in ab initio molecular dynamics (AIMD) simulations (NVT ensemble), which is carried out for 3 ps with a time step of 1 fs at 500 K (Cimas et al., 2014).

The optical properties can be defined by the complex dielectric function (frequency) for characterization:

$$\varepsilon \left(\omega \right)=\varepsilon_{1}\left(\omega \right)+i\varepsilon_{2}\left(\omega \right)$$ (1)

where $\varepsilon_{1}\left(\omega \right)$ and $\varepsilon_{2}\left(\omega \right)$ represent the real and imaginary parts, respectively. Based on the Kramers–Kronig transformation (Kuzmenko, 2005), the real part $\left[\varepsilon_{1}\left(\omega \right)\right]$ can be expressed as follows:

$$\varepsilon_{1}\left(\omega \right)=1+\left(\frac{2}{\pi }\right)p\int_{0}^{\infty }d\omega^{\prime }\frac{{\left(\omega^{\prime }\right)}^{2}\varepsilon_{2}\left(\omega \right)}{{\left(\omega^{\prime }\right)}^{2}-{\left(\omega \right)}^{2}}$$ (2)

where p is the integral principal value. In addition, the imaginary part $\left[\varepsilon_{2}\left(\omega \right)\right]$ can be described as (Saha et al., 2000):

$$\varepsilon_{2}\left(\omega \right)=\frac{4\pi e^{2}}{m^{2}\omega^{2}}\sum_{i,f}\int \frac{2d^{3}k}{{\left(2\pi \right)}^{3}}{\left|\langle ik|P|fk\rangle \right|}^{2}F_{i}^{k}\left(1-F_{f}^{k}\right)\delta \left(E_{f}^{k}-E_{i}^{k}-E\right)$$ (3)

where ω , E , F , P , ||ik〉 , and |fk〉 represent the incident photon frequency, the incident photon energy, the Fermi function, the transition matrix, the CB state and VB state, respectively. Finally, the absorption coefficient [α(ω)] can be calculated by (Cheng et al., 2022a):

$$\alpha \left(\omega \right)=\frac{\sqrt{2\omega }}{c}{\left[\sqrt{\varepsilon_{1}^{2}\left(\omega \right)+\varepsilon_{2}^{2}\left(\omega \right)}-\varepsilon_{1}\left(\omega \right)\right]}^{1/2}$$ (4)

where c denotes the speed of light in vacuum.

The carrier mobility is calculated by the following formula (Qiao et al., 2014):

$$\mu =\frac{e\hbar^{3}C_{2D}}{k_{B}Tm_{e}^{*}m_{d}E_{d}^{2}}$$ (5)

where $C_{2D}$ is the elastic modulus, which can be expressed by $C_{2D}=\frac{1}{S_{0}}\frac{\partial^{2}E}{\partial \delta^{2}},$ here E is the total energy of monolayer after deformation, $S_{0}$ is the lattice area of monolayer under equilibrium and δ is the uniaxial strain. Besides, $k_{B}$ is the Boltzmann constant, T is the temperature. $m_{e}^{*}$ is the effective mass along the transport direction and $m_{d}$ is the average effective mass determined by $m_{d}=\sqrt{m_{x}^{*}m_{y}^{*}}$ . $E_{d}$ represents the deformation potential constant, which is expressed by $E_{d}=\frac{\partial E_{edge}}{\partial \delta }$ , here $E_{edge}$ represents the shift of the band edge position with respect to the uniaxial strain δ .

## Results and discussions

### Crystal structures, stability, and electronic properties

**FIGURE 1:**
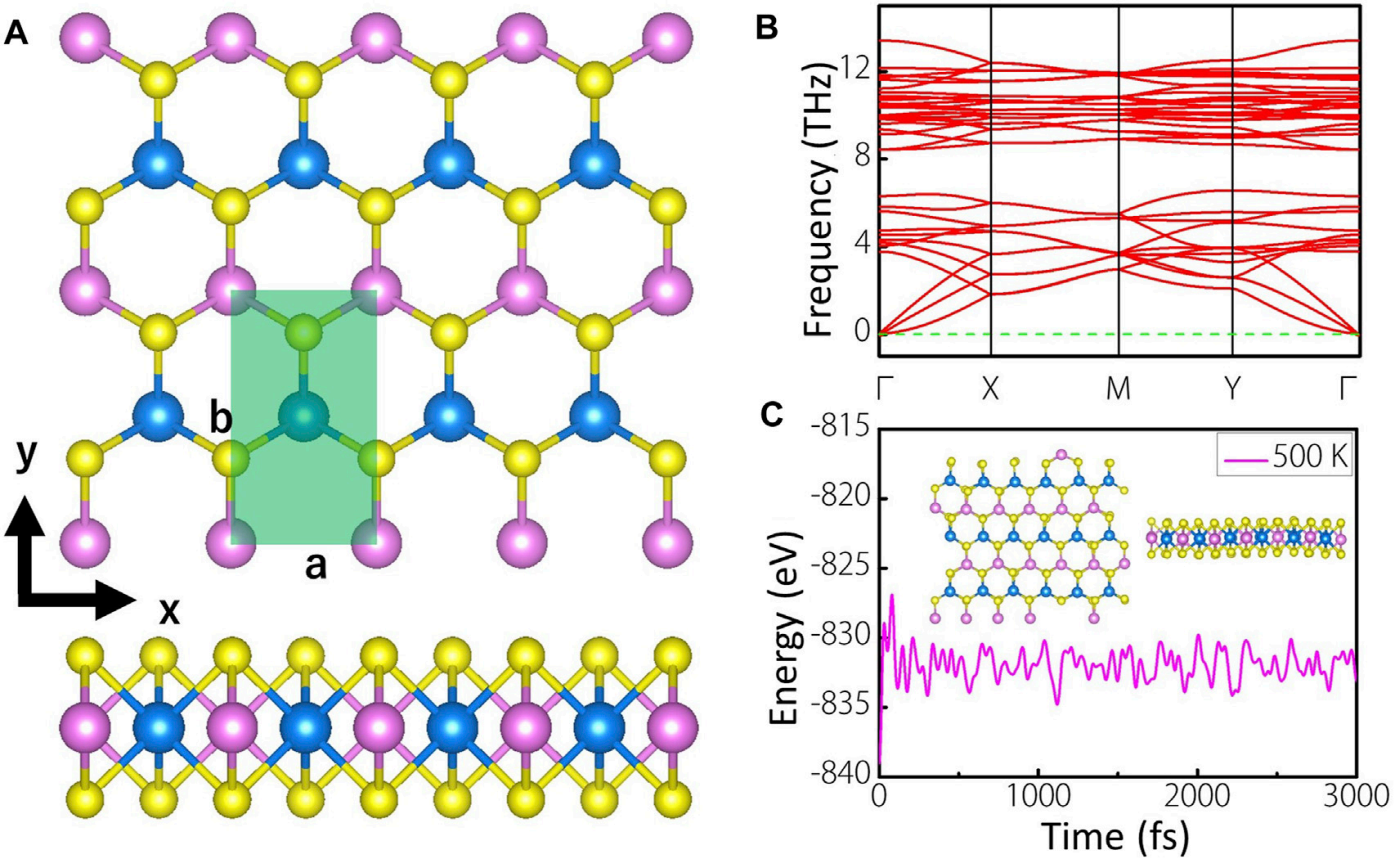
**(A)** Top and side views of MoWS_4_ monolayer. The pink, blue, and yellow balls represent Mo, W, and S atoms, respectively. The light green area is the unit cell of MoWS_4_ monolayer **(B)** Phonon spectra of MoWS_4_ monolayer **(C)** Evolution of the total energy and a snapshot of MoWS_4_ monolayer after a 3,000 fs AIMD simulations in vacuum at 500K

Figure 1A shows the top and side views of 4 × 2 supercell of MoWS_4_ monolayer, which has a space group of *Pmm2*. The light green area is the unit cell of MoWS_4_ monolayer, which contains four atoms. The pink, blue, and yellow balls represent Mo, W, and S atoms, respectively. MoWS_4_ monolayer is a honeycomb structure from the top view, while sandwich structure from the side view, which is very similar to the structure of MoS_2_ monolayer (Kan et al., 2014). After fully geometric optimization, the lattice constants are *a* = 3.188 Å and *b* = 5.526 Å with a layer thickness of 3.119 Å.

To confirm the stability of MoWS_4_ monolayer, the formation energy ( $E_{form}$ ), defined as: $E_{form}=\left[E_{MoWS_{4}}-E_{Mo}-E_{W}-4E_{S}\right]/6$ , is calculated, where $E_{MoWS_{4}}$ presents the total energy of the unit cell of MoWS_4_ monolayer, and $E_{Mo}$ , $E_{W}$ , and $E_{S}$ present the energies of each Mo, W, and S atom in itself bulk phase, respectively. The calculated formation energy of MoWS_4_ monolayer is −1.1 eV/atom (<0), implying the procedure of synthesizing MoWS_4_ monolayer is exothermic and favorable. Besides, the dynamic and thermal stabilities of MoWS_4_ monolayer are further investigated. As shown in Figure 1B, the calculated phonon spectra exhibit no imaginary frequency, indicating high dynamic stability of MoWS_4_ monolayer. The thermal stability is further studied *via* AIMD calculations. As illustrated in Figure 1C, the total energy of the MoWS_4_ monolayer remains essentially stable and no deformation occurs in its final structure. These results suggest that MoWS_4_ monolayer exhibits good dynamic and thermal stability, which deduces the possibility of experimental synthesis of MoWS_4_ monolayer.

**FIGURE 2:**
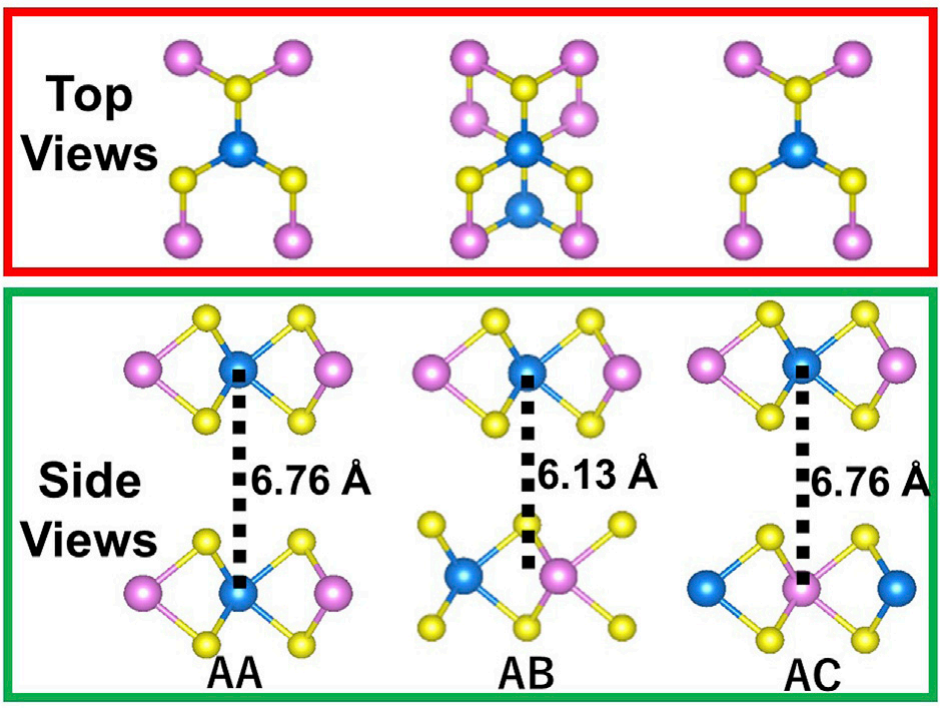
Top and side views of MoWS_4_ bilayer with three stacking patterns (AA, AB, and AC).

Figure 2 shows the top and side views of MoWS_4_ bilayer. Three stacking patterns (AA, AB, and AC) are considered in the bilayer structures of MoWS_4_. The results of the calculated total energy are −94.2727 eV, −94.4175 eV, and −94.2726 eV for AA, AB, and AC stacking patterns, respectively. AA and AC stacking structures have the same layer spacing of 6.76 Å, and AB stacking has a spacing of 6.13 Å. Obviously, the AB stacking structure is most stable energetically.

**FIGURE 3:**
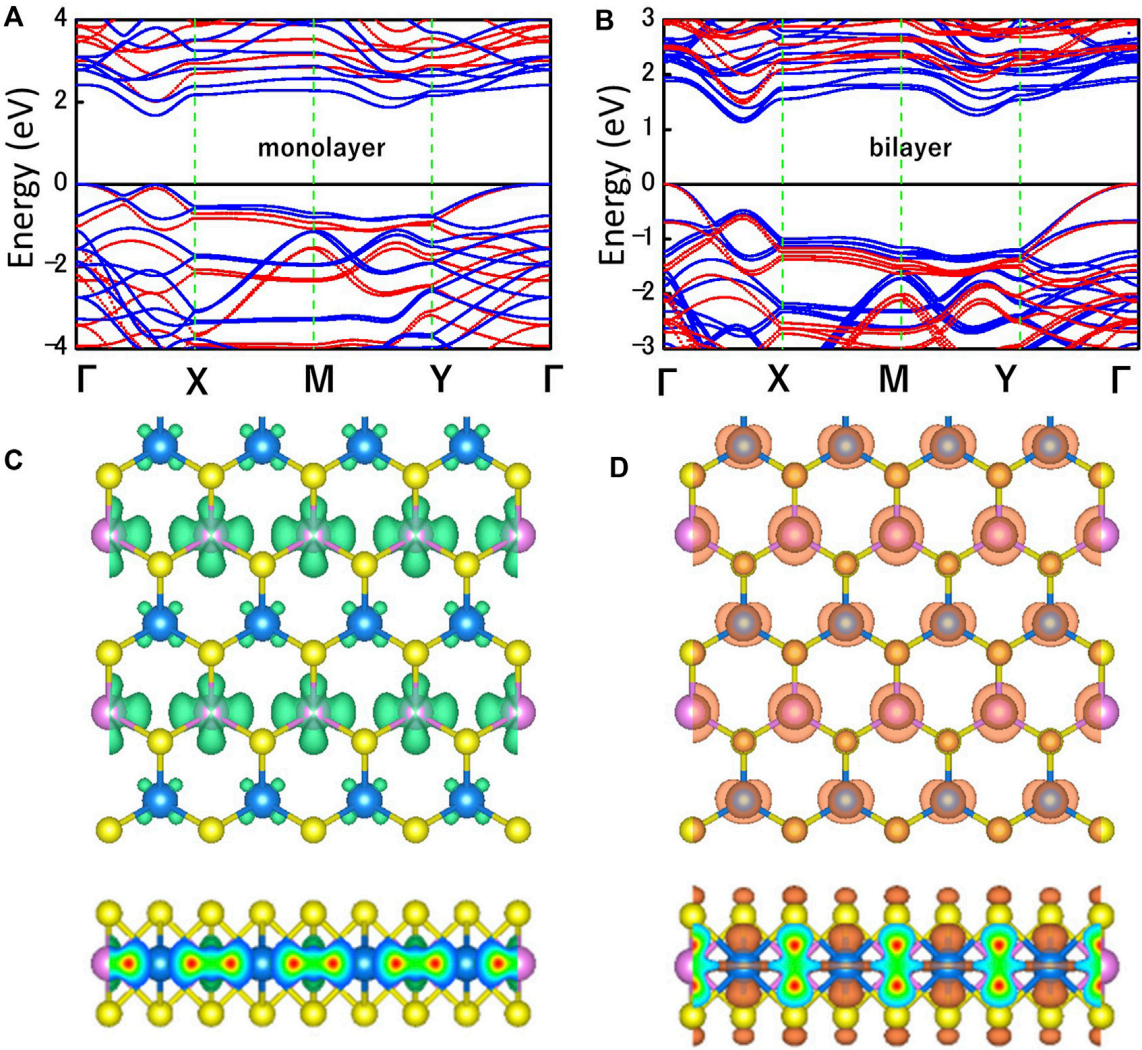
Band structures of **(A)** MoWS_4_ monolayer and **(B)** MoWS_4_ bilayer. The red and blue represent the HSE06 functional and PBE functional, respectively. Top and side views of the charge density at the **(C)** CBM, and **(D)** VBM. The isosurface value is set as 0.012 e Å^−3^.

Figures 3A,B shows the electronic band structures of MoWS_4_ monolayer and bilayer obtained by using the PBE functional (blue) and the HSE06 functional (red). The Fermi level and the high symmetry path are set as 0 eV and *Γ-X-M-Y-Γ*, respectively. The MoWS_4_ monolayer and bilayer are indirect band gap semiconductors and their band gaps calculated by HSE06/PBE functional are 2.01/1.67 eV and 1.48/1.14 eV, respectively. The band gaps obtained by the HSE06 functional are larger than that obtained by the PBE functional since the PBE functional tends to underestimate the band gap. Figures 3C,D shows the maps of charge density for MoW_4_ monolayer at the conduction band minimum (CBM) and the valence band maximum (VBM), and it can be concluded that the CBM is mainly contributed by the Mo atoms, whereas the VBM comes from a combination of Mo, W, and S atoms.

### Photocatalytic water splitting and optical properties

**FIGURE 4:**
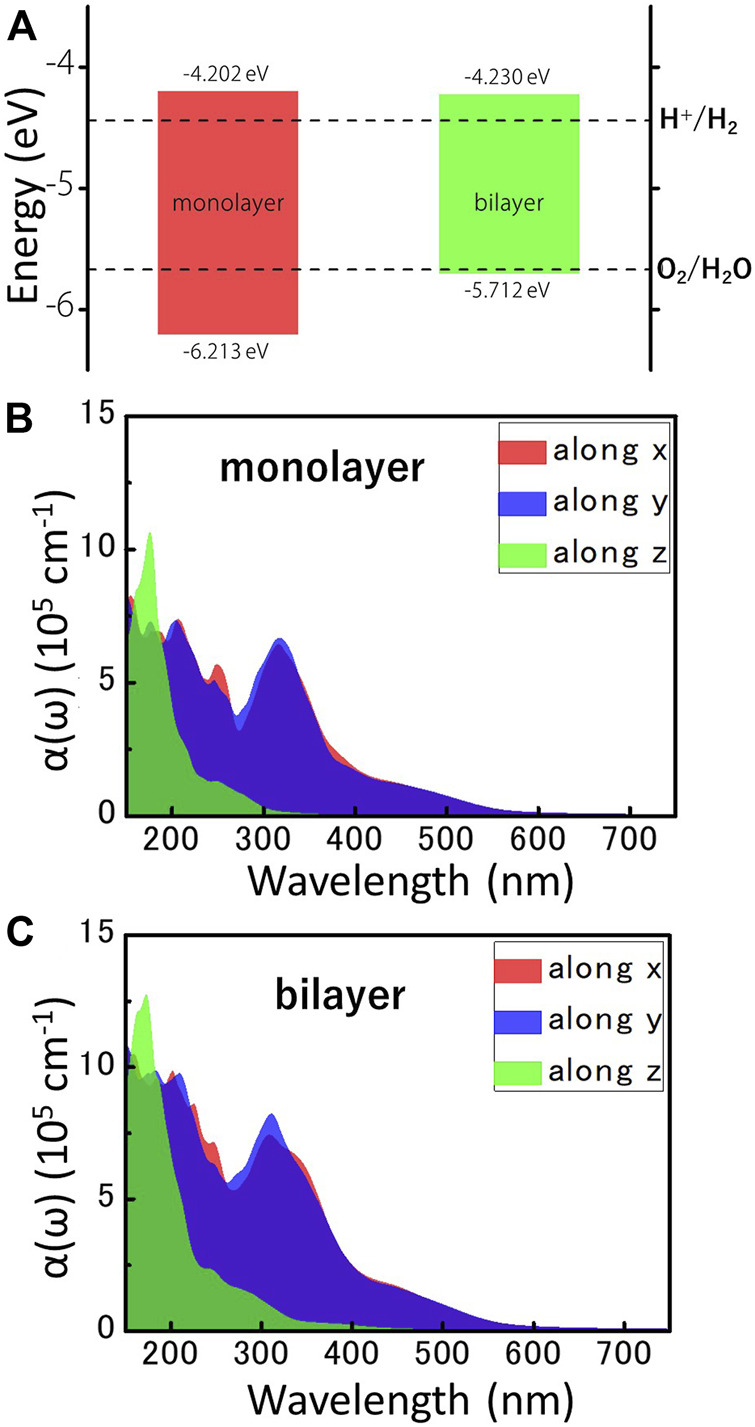
**(A)** The locations of VBM and CBM of MoWS_4_ monolayer and bilayer, which are obtained by the HSE06 functional. The redox potentials of water splitting at pH = 0 are shown for comparison. All the energy levels are based on the vacuum level, and the vacuum level is set as 0 eV. The optical absorption coefficients for the different directions of **(B)** MoWS_4_ monolayer and **(C)** MoWS_4_ bilayer.

Considering the excellent semiconductor properties of MoWS_4_ monolayer and bilayer, we systematically study their feasibility as photocatalysts for water splitting. It is well known that a photocatalytic candidate should meet the following conditions: Firstly, its band gap should exceed the free energy of water splitting (1.23 eV). Obviously, the band gaps of MoWS_4_ monolayer and bilayer both exceed 1.23 eV; Secondly, its band edges must cross the redox potentials of water. The CBM energy should be higher than the reduction potential of H^+^/H_2_ (−4.44 eV), and the VBM energy should be lower than the oxidation potential of O_2_/H_2_O (−5.67 eV) (Abe, 2010; Sun et al., 2019; Cheng et al., 2022b). For 2D materials, the band edges with respect to the vacuum level ( $E_{CBM/VBM}^{Vac}$ ) can be obtained by: $E_{CBM/VBM}^{Vac}=E_{CBM/VBM}^{DFT}-V_{vacuum}$ , where $E_{CBM/VBM}^{DFT}$ represents the value of CBM/VBM obtain by DFT and $V_{vacuum}$ represents the electrostatic potential in the vacuum region. We plot the map of band edges relative to the vacuum level of MoWS_4_ monolayer and bilayer in Figure 4A, and find that the band edge positions of MoWS_4_ monolayer and bilayer can perfectly satisfy the redox potential for the water splitting reaction at pH = 0. Specifically, the band edges of CBM/VBM for MoWS_4_ monolayer and bilayer are −4.202/−6.213 eV and −4.230/−5.712 eV, respectively. Thus, the results suggest that the MoWS_4_ monolayer and bilayer can be promising candidates for water splitting photocatalysts.

Besides, efficient light absorption is also an important feature for water splitting photocatalysts. Figures 4B,C shows the light absorption spectra of the MoWS_4_ monolayer and bilayer within the visible-ultraviolet light range. The result can be summarized as follows: 1) Their light absorption coefficients in the ultraviolet light range are higher than visible light region (up to ∼10^6^ cm^−1^); 2) Their light absorption coefficients of MoWS_4_ bilayer overall are higher than that of MoWS_4_ monolayer; 3) Their light absorption coefficients are significantly anisotropic: that is higher in the *z* direction, but have a wider range of light absorption spectra in the *x* and *y* directions. It is known that the high light absorption coefficients can guarantee the effective use of solar energy, which is very favorable for water splitting photocatalysts (Zhao et al., 2018; Fan et al., 2021). Therefore, both MoWS_4_ monolayer and bilayer can be promising potential candidates as photocatalysts.

### Strain engineering and high carrier mobility

**FIGURE 5:**
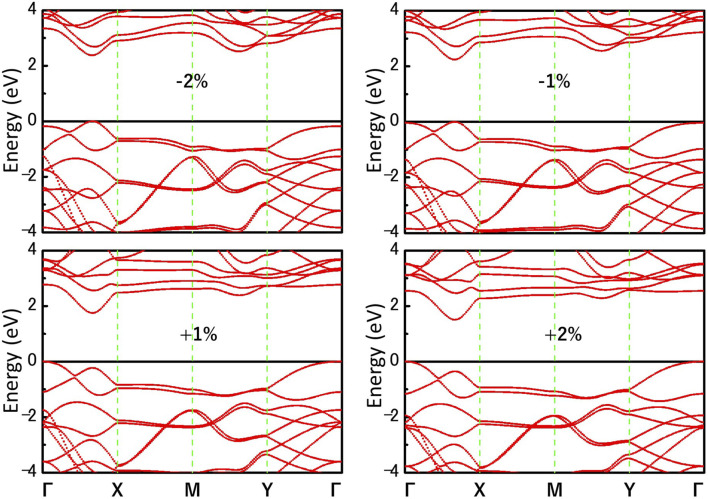
Band structures of MoWS_4_ monolayer under the biaxial strain from −2 to 2% are calculated by HSE06 functional.

**FIGURE 6:**
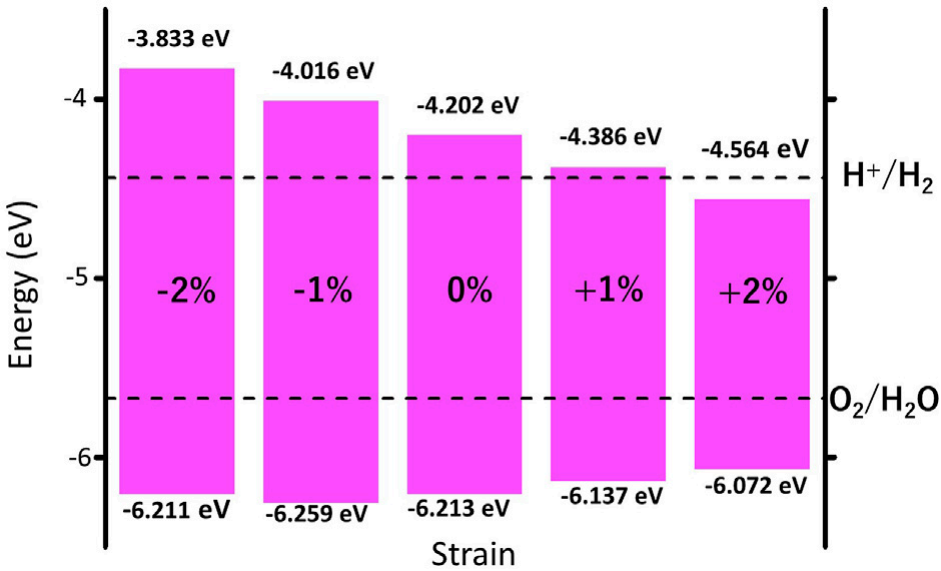
Strain effects on band edge positions of MoWS_4_ monolayer with respect to the vacuum level (0 eV). The redox potentials of water splitting at pH = 0 are shown for comparison.

We further investigate the effect of strain engineering on the photocatalytic performance of MoWS_4_ monolayer. Figure 5 shows the band structures of MoWS_4_ monolayer under the in-plane biaxial strain from −2 to 2%. Its band gap increases when compressive strain increases or tensile strain decreases, and its band gap varies in the range of 1.508–2.378 eV. In addition, when it is subjected to compressive strain, the indirect bandgap changes to be a direct bandgap; while when it is subjected to tensile strain, it remains an indirect bandgap semiconductor. As illustrated in Figure 6, we study the changes of its band edge positions under the applied in-plane biaxial strain. All of VBM positions are lower than the oxidation potential of O_2_/H_2_O under the −2∼2% in-plane biaxial strain, which indicates that MoWS_4_ monolayer always serves as a potential photocatalyst to generate oxygen. Besides, when the applied tensile strain reaches 2%, its CBM positions become lower than the reduction potential of H^+^/H_2_. Thus, excessive tensile strain will lead to its inability to produce hydrogen. In general, compressive strain does not cause MoWS_4_ monolayer to deviate from the basic requirements for water splitting photocatalyst, but tensile strain can easily affect its hydrogen production performance.

**FIGURE 7:**
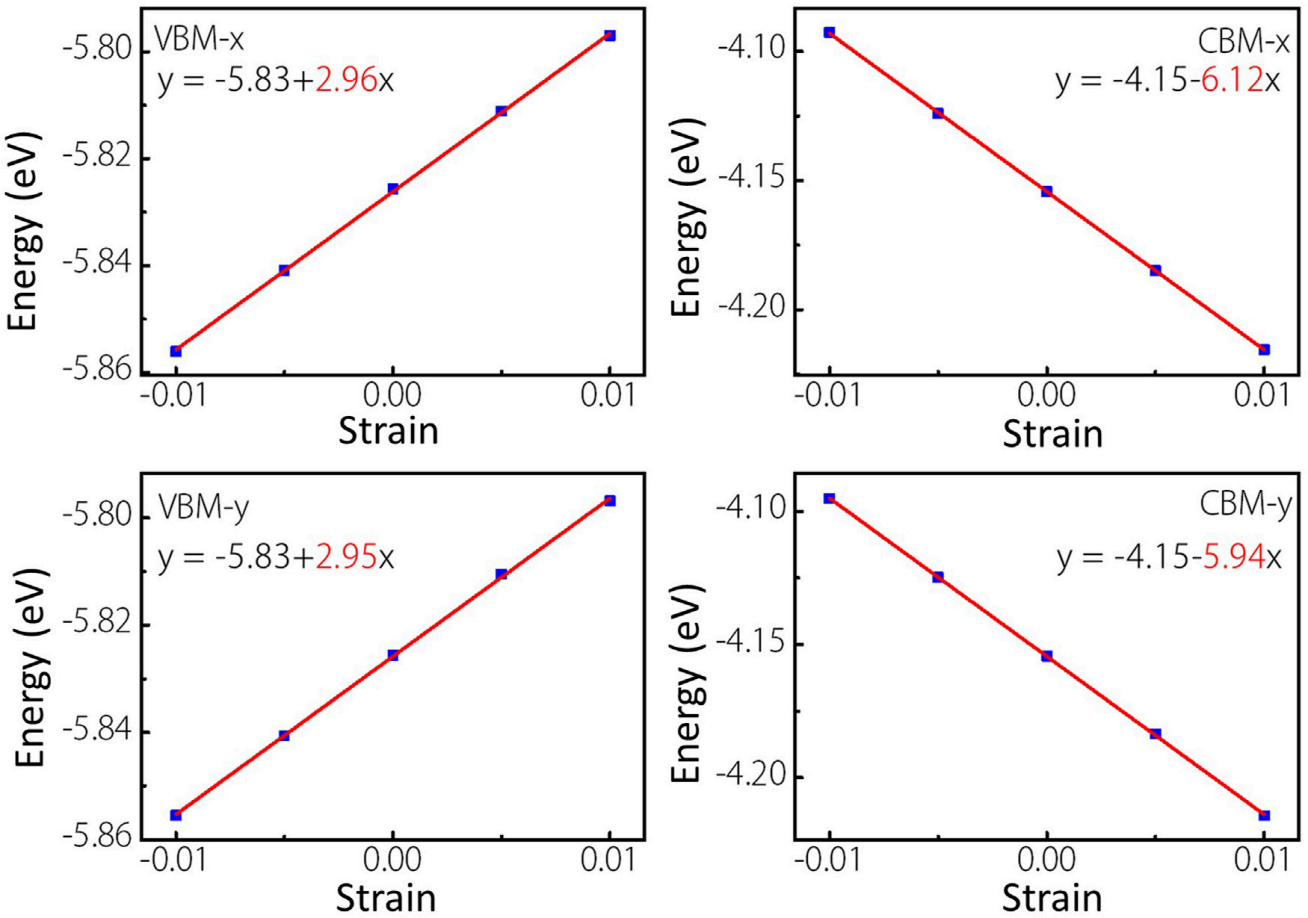
Linear fitting maps of VBM and CBM locations of MoWS_4_ monolayer under the strain along *x* and *y* directions.

**FIGURE 8:**
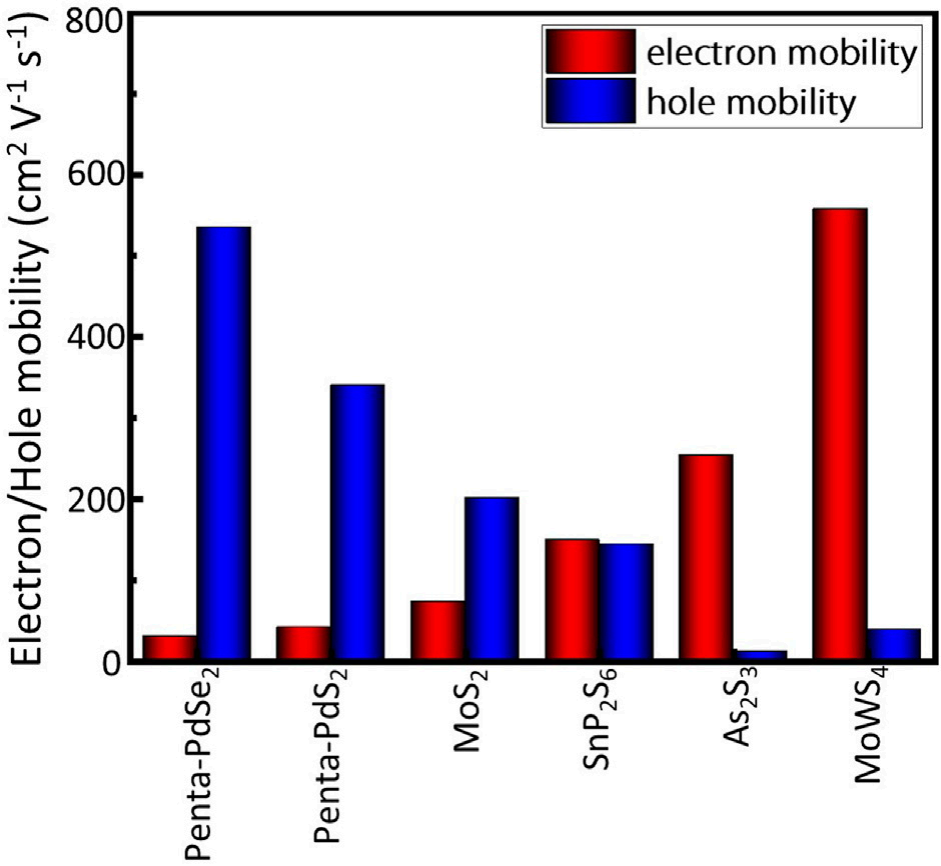
Carrier mobility of MoWS_4_ compared with other typical 2D photocatalysts.

As we know, the fast carrier migration capability is necessary for high performance photocatalysts. Thus, the PBE functional is used to calculate the carrier mobility of MoWS_4_ monolayer according to Eq. 5. Subsequently, we calculate its carrier effective masses $\left(m^{*}\right)$ , in-plane stiffness $\left(C_{2D}\right)$ and deformation potential constants $\left(E_{d}\right)$ . Therein, to obtain $E_{d}$ of MoWS_4_ monolayer, the linear fitting maps of band edge positions are plotted as the function of the applied uniaxial strain δ along the *x* and *y* directions (Bardeen and Shockley, 1950; Zhang et al., 2021a), as illustrated in Figure 7 and the result is summarized in Table 1. It is notable that the values of $E_{d}$ have a small difference along the *x* and *y* directions, which shows that the scattering ability of its carriers in different directions is also similar. In addition, the carrier effective masses of MoWS_4_ monolayer have a small difference along the *x* and *y* directions. The electron effective masses of MoWS_4_ monolayer are much lower than its hole effective masses (*m*_*e*_ = 0.377*m*_*0*_ and 0.374*m*_*0*_ along the *x* and *y* directions), which are smaller than that of many 2D photocatalytic materials, such as Penta-PdSSe (*m*_*e*_ = 2.16*m*_*0*_), Penta-PdSe_2_ (*m*_*e*_ = 1.88*m*_*0*_), δ-SnS (*m*_*e*_ = 1.01*m*_*0*_), SnP_3_ (*m*_*e*_ = 0.9*m*_*0*_) (Long et al., 2018; Sun et al., 2018; Zhang et al., 2021b; Xiao et al., 2021). The smaller carrier effective masses benefit the transfer rate of photogenerated carriers in the photocatalytic process. What’s more, the calculated electron mobility along *x* and *y* directions are 529 cm^2^V^−1^s^−1^ and 557 cm^2^V^−1^s^−1^, respectively, whereas the calculated hole mobility along *x* and *y* directions are both 38 cm^2^ V^−1^s^−1^. The reason for this difference is that the hole effective mass is much larger than the electron effective mass. More importantly, as shown in Figure 8, the electron mobility of MoWS_4_ monolayer can significantly exceed that of many other 2D photocatalytic materials such as MoS_2_ (*µ*_*e*_ = 72.16 cm^2^ V^−1^ s^−1^, *µ*_*h*_ = 200.52 cm^2^ V^−1^ s^−1^), Penta-PdS_2_ (*µ*_*e*_ = 40.97 cm^2^ V^−1^ s^−1^, *µ*_*h*_ = 339.25 cm^2^ V^−1^ s^−1^), Penta-PdSe_2_ (*µ*_*e*_ = 29.40 cm^2^ V^−1^ s^−1^, *µ*_*h*_ = 534.55 cm^2^ V^−1^ s^−1^), SnP_2_S_6_ monolayer (*µ*_*e*_ = 148.48 cm^2^ V^−1^ s^−1^, *µ*_*h*_ = 143.12 cm^2^ V^−1^ s^−1^), As_2_S_3_ monolayer (*µ*_*e*_ = 253.11 cm^2^ V^−1^ s^−1^, *µ*_*h*_ = 10.85 cm^2^ V^−1^ s^−1^) (Cai et al., 2014; Wang et al., 2015; Long et al., 2018; Jing et al., 2019; Liu et al., 2021). It is well known that materials with high carrier mobility can effectively reduce their photogenerated electron and hole recombination rates and increase the participation rate in redox reactions, which are beneficial for photocatalytic processes. Thus, our results suggest that MoWS_4_ monolayer is a promising 2D photocatalyst candidate for water splitting.

**TABLE 1:** Carrier effective masses $m^{*}$ , In-plane stiffness $C_{2D}$ , Deformation potential constants $E_{d}$ , and carrier mobility μ for MoWS_4_ monolayer along the *x* and *y* directions.

Materials	Direction	Carrier type	$m^{*}$ ( $m_{0}$ )	$C_{2D}$ (N/m)	$E_{d}$ (eV)	μ (cm^2^V^−1^s^−1^)
MoWS_4_	*X*	Electron	0.377	131.86	6.12	529
		Hole	2.927	131.86	2.96	38
	*Y*	Electron	0.374	129.5	5.94	557
		Hole	2.857	129.5	2.95	38

## Conclusion

Based on the first-principles calculations, we systematically investigate photocatalytic properties of two-dimensional MoWS_4_. It is found that both the MoWS_4_ monolayer and bilayer are semiconductors with indirect band gaps and show high and anisotropic light absorption coefficients in the visible-ultraviolet range. The band edge positions of the materials can satisfy the redox potentials perfectly and the electron mobility of MoWS_4_ monolayer is up to 557 cm^2^ V^−1^ s^−1^, which outperforms many other 2D photocatalytic materials, such as MoS_2_ monolayer, Penta-PdS_2_, Penta-PdSe_2_, and SnP_2_S_6_ monolayer. These results indicate that MoWS_4_ can be a promising photocatalyst for water splitting with outstanding performances.

## Data Availability

The original contributions presented in the study are included in the article/Supplementary Material, further inquiries can be directed to the corresponding authors.
